# Longitudinal Association of Depression Symptoms With Cognition and Cortical Amyloid Among Community-Dwelling Older Adults

**DOI:** 10.1001/jamanetworkopen.2019.8964

**Published:** 2019-08-09

**Authors:** Jennifer R. Gatchel, Jennifer S. Rabin, Rachel F. Buckley, Joseph J. Locascio, Yakeel T. Quiroz, Hyun-Sik Yang, Patrizia Vannini, Rebecca E. Amariglio, Dorene M. Rentz, Michael Properzi, Nancy J. Donovan, Deborah Blacker, Keith A. Johnson, Reisa A. Sperling, Gad A. Marshall

**Affiliations:** 1 Division of Geriatric Psychiatry, McLean Hospital, Harvard Medical School, Belmont, Massachusetts; 2 Department of Psychiatry, Massachusetts General Hospital, Harvard Medical School, Boston; 3 now with Sunnybrook Research Institute, Toronto, Ontario, Canada; 4 Department of Neurology, Massachusetts General Hospital, Harvard Medical School, Boston; 5 Florey Institutes of Neuroscience and Mental Health, Melbourne, Australia; 6 Center for Alzheimer Research and Treatment, Department of Neurology, Brigham and Women’s Hospital, Harvard Medical School, Boston, Massachusetts; 7 Melbourne School of Psychological Sciences, University of Melbourne, Melbourne, Australia; 8 Department of Radiology, Massachusetts General Hospital, Boston; 9 Division of Geriatric Psychiatry, Brigham and Women’s Hospital, Harvard Medical School, Boston, Massachusetts; 10 Department of Epidemiology, Harvard T.H. Chan School of Public Health, Boston, Massachusetts

## Abstract

**Importance:**

Depressive symptoms are prevalent among older adults and may be early manifestations of Alzheimer disease (AD) before onset of mild cognitive impairment. However, it remains unclear whether worsening depressive symptoms in the presence of AD pathology are associated with cognitive decline in older adults.

**Objective:**

To determine the longitudinal association between depressive symptoms, cognition, and cortical amyloid in community-dwelling older adults.

**Design, Setting, and Participants:**

Participants from the Harvard Aging Brain Study, a cohort study, underwent annual assessments of depression and cognition and baseline cortical amyloid measurement (mean, 4.42 years; range, 2-7 years). Data collection was from September 2010 to August 2017 in a convenience sample of community-dwelling adults (276 participants, all cognitively unimpaired) with at most mild depression at entry.

**Main Outcomes and Measures:**

Depression (Geriatric Depression Scale [GDS]), cognition (Preclinical Alzheimer Cognitive Composite [PACC]), and a continuous measure of cortical amyloid (Pittsburgh Compound-B positron emission tomography imaging). Change in GDS and baseline amyloid were examined as interactive predictors of PACC decline in a linear mixed model with backward elimination, adjusting for age, sex, and education.

**Results:**

Participants were 164 women and 112 men (mean [SD] age, 73.5 [6.0] years). At baseline, the mean (SD) GDS score was 3.0 (2.8) (range, 0-12), the mean (SD) PACC score was −0.004 (0.67) (range, −2.32 to 1.88), and the mean (SD) amyloid positron emission tomography distribution volume ratio was 1.16 (0.20) (range, 0.92-1.94). At last follow-up, the mean (SD) GDS score was 3.9 (2.9) (range, 0-12), and the mean (SD) PACC score was −0.09 (1.27) (range, −5.66 to 1.67). The interaction between cortical amyloid and increasing GDS was associated with declining cognition (β = −0.19; 95% CI, −0.27 to −0.12; *P* < .001).

**Conclusions and Relevance:**

In this study, cortical amyloid moderated the association between worsening depressive symptoms and declining cognition in older adults. While future work is needed to better understand causal associations, these findings may enhance early detection and prevention of AD clinical symptoms.

## Key Points

**Question:**

Do changing depressive symptoms over time in the presence of cortical amyloid—an in vivo marker of Alzheimer disease pathology—relate to changes in cognition in older adults?

**Findings:**

In longitudinal data from a cohort study of 276 older adults, all of whom were cognitively unimpaired and had at most mild depression at study entry, worsening depressive symptoms over 2 to 7 years in the presence of cortical amyloid were significantly associated with declining cognition.

**Meaning:**

Concurrent changes in depression and cognition among older adults with higher cortical amyloid suggest that depressive symptoms may serve as targets in delaying the clinical symptoms of Alzheimer disease.

## Teaser

This cohort study investigates the longitudinal association between depressive symptoms, cognition, and cortical amyloid in unimpaired community-dwelling older adults in the Harvard Aging Brain Study.

## Introduction

Depressive symptoms, including those in the subthreshold and mild range, are prevalent and distressing among older adults. Such symptoms might also serve as clinical indicators or modifiers of cognitive performance and Alzheimer disease (AD).

Increasingly, AD observational and intervention research has focused on the preclinical stage of AD, in which there is evidence of elevated amyloid and pathological change well before the development of mild cognitive impairment (MCI).^1,2,3^ Interventions at this preclinical stage may have the potential to prevent subsequent decline.^4^ Secondary prevention trials in preclinical AD rely on sensitive measures of cognition weighted toward episodic memory to detect subtle changes in cognitive performance before there is clearly defined cognitive impairment.^5^ Such trials now focus on cognitive outcomes and do not incorporate depression as an outcome measure.

Previous work has shown that depressive and mood symptoms in cognitively unimpaired older adults may be associated with amyloid and tau,^6,7,8,9^ hallmarks of AD pathology that can be measured in vivo. Prior work has also established an association between depressive symptoms and multidomain cognitive deficits in older adults.^10,11^ However, the association between mild depressive symptoms and cognition in the presence of in vivo AD pathology in unimpaired older adults has not been clearly established. Better understanding of the associations among depressive symptoms, cognition, and AD pathology is critical to inform prognosis in older adults with depressive symptoms, some of whom may be at risk for cognitive decline. Increased understanding also has relevance for preclinical AD prevention trials, in which depressive symptoms could serve as viable targets in delaying the clinical symptoms of AD.

In this study, we sought to determine whether baseline cortical amyloid modified the longitudinal association between depressive symptoms and cognition in unimpaired older adults in the Harvard Aging Brain Study (HABS). HABS is an observational cohort study of cognitive aging and preclinical AD that includes multimodal neuroimaging and annual clinical assessments.^12,13^ Previous HABS investigations have shown associations between baseline cortical amyloid and longitudinally increasing depressive symptoms^8^ and between baseline amyloid and declining cognition.^14^ To build on these prior findings, we chose to focus on how—in the setting of cortical amyloid—changes in depressive symptoms over time are associated with changes in cognitive performance. We hypothesized that cortical amyloid would moderate the association between depressive symptoms and cognitive decline, such that the interaction between amyloid and increasing depressive symptoms would be associated with greater cognitive decline. Any observed associations would underscore the need for close clinical follow-up of older adults with depressive symptoms and AD pathology, who may be at heightened risk for cognitive decline.

## Methods

### Study Sample

Participants in HABS are English-speaking community-dwelling older adults. All participants were cognitively unimpaired at study entry, having a global Clinical Dementia Rating^15^ of 0 and a Mini-Mental State Examination^16^ of at least 27 with educational adjustment (for low education, scores ≥25 were permitted).^17^ In addition, all performed in the normal range within education-adjusted norms on the Logical Memory IIa Delayed Recall Index from the Wechsler Memory Scale.^18^ Exclusion criteria at HABS entry included history of neurological disorders and history of psychiatric disorders (ie, schizophrenia, schizoaffective disorder, and bipolar disorder). Substance use disorder within the past 2 years and unstable medical conditions were additional exclusions. Participants were permitted to have current mild depression or anxiety symptoms (ie, 30-item Geriatric Depression Scale [GDS] score ≤12) and be receiving stable treatment (≥30 days), including selective serotonin reuptake inhibitors or selective serotonin-norepinephrine reuptake inhibitors, bupropion, or nortriptyline hydrochloride. While a GDS greater than 12 was an exclusion criterion at HABS entry, those who developed scores above this on subsequent annual follow-up visits continued to be followed.

From among the HABS cohort, we included all individuals for the present analyses who had at least 2 years of cognitive data (mean, 4.4 years per participant; minimum, 2 years; and maximum, 7 years) and had completed baseline amyloid positron emission tomography (PET) imaging; a total of 276 individuals met these criteria. The number of participants with a given amount of follow-up is listed in eTable 1 in the Supplement. As per the HABS protocol, participants underwent baseline amyloid PET imaging and annual assessments of depression and cognition (mean, 4.42 years; range, 2-7 years). Data collection was from September 2010 to August 2017. The HABS protocol is approved annually by the Partners Human Research Committee. All participants provided written informed consent before any study procedures.

Further details of HABS have been described elsewhere.^19^ The present study followed the Strengthening the Reporting of Observational Studies in Epidemiology (STROBE) reporting guideline for cohort studies.

### Depression Assessment

Depression was assessed using the GDS; higher scores on this 30-item (yes or no) self-report scale indicate greater depression.^20^ Across the longitudinal data set, about 3% of GDS records contained 1 or more missing items (usually only 1 item was missing). For cases in which 3 or fewer items for a GDS record were missing (n = 41), responses on the remaining items were averaged and extrapolated to a maximum 30-point possible total scale score for comparability with other GDS results (eAppendix 1 in the Supplement). Antidepressant medication use at baseline was obtained by self-report and was included in secondary analyses as “yes or no” if the participant reported use of a selective serotonin reuptake inhibitor, a selective serotonin-norepinephrine reuptake inhibitor, or another class of antidepressant. History of depression and time of onset of first episode were obtained by self-report at the initial visit. Participants were categorized as having no depression history or as having history with recent onset (within the past 10 years) or more remote onset (>10 years before study entry).

### Neuropsychological Evaluation

All participants underwent annual evaluation with the Preclinical Alzheimer Cognitive Composite (PACC), a composite weighted toward episodic memory that is sensitive to cognitive decline in preclinical AD.^5^ The PACC is a continuous measure composed of the following: Mini-Mental State Examination,^16^ Logical Memory IIa Delayed Recall Index,^18^ the Digit Symbol Substitution Test from the Wechsler Adult Intelligence Scale–Revised,^21^ and Free and Cued Selective Reminding (sum of free recall plus total recall).^22^ To compute the PACC, we calculated the *z* score for each test based on the mean (SD) of the baseline scores and then took the mean *z* score across all 4 tests. A higher PACC indicates better cognition.

### Pittsburgh Compound-B PET Imaging

Baseline cortical amyloid retention values derived from Pittsburgh Compound-B (PiB) PET scans completed during year 1 of HABS were used for all analyses. Given the slow rate of change of PiB over the mean period of interest (1-4 years), baseline PiB values were used as representative measures of amyloid burden.

Synthesis and administration of carbon 11–labeled PiB took place at Massachusetts General Hospital on an ECAT EXACT HR+ scanner (Siemens) according to previously described methods.^23^ Distribution volume ratios (DVRs) were developed from a global cortical aggregate of the frontal, lateral, and retrosplenial regions given that neocortical regions have a high degree of collinearity in amounts of amyloid deposition. The PiB DVR values were normalized to the reference region (cerebellar gray) derived from the FreeSurfer atlas.^24^

### Statistical Analysis

Analyses were mixed-effects longitudinal models with backward elimination. In primary analyses, change in GDS and baseline amyloid were examined as interactive predictors of PACC decline in a linear mixed model with backward elimination, adjusting for age, sex, and education. The threshold for statistical significance was *P* < .01. Hypothesis tests were 2-sided. All analyses were performed using the software program SAS (version 9.4; SAS Institute Inc) or R (version 3.2.4; R Foundation).

#### Association of Longitudinal PACC and Longitudinal GDS

To test our hypothesis of primary interest—that PACC and GDS were inversely associated over the course of the study in the presence of baseline amyloid—we ran a mixed-effects model in which PACC (outcome) was predicted by longitudinal GDS and baseline cortical amyloid (PiB PET). Initial fixed terms were baseline age, sex, years of education, longitudinal GDS (linear and quadratic), baseline amyloid (PiB PET), longitudinal GDS by baseline amyloid interaction, and longitudinal GDS by covariates interaction. Random terms were participant intercept and longitudinal GDS (random linear term for the slope of the association between GDS and PACC ). For this and all other models, we used a backward elimination approach with a *P* < .01 cutoff, in which a few higher-order terms (ie, quadratic terms and interactions) were initially included. Higher-order terms with *P* values above this threshold were eliminated from the model (eAppendix 2 in the Supplement).

To determine whether GDS had a negative association with PACC at baseline PiB levels below the published threshold of 1.20 DVR that typically marks amyloid positivity in HABS,^25^ we computed the value of PiB at which the regression coefficients of the interaction term (GDS by PiB) and main association term for GDS summed to zero, as per previously described methods.^26^ This value is synonymous with the value of PiB at which the slope of GDS and PACC changed direction and became negative.

We adjusted for the potentially confounding association of time (ie, to rule out an association between GDS and PACC across the study due to each being independently related to time but otherwise unrelated to each other). To do so, the primary model (a linear mixed model with backward elimination in which change in GDS and baseline amyloid were examined as interactive predictors of PACC decline, adjusting for age, sex, and education) was repeated holding time constant and including covariates with and without their interactions with time.

#### Exploratory Analysis of Time Lag Associations

To better understand the direction and temporal associations between changes in GDS and in PACC, we ran a series of mixed-effects cross-lagged analyses^27^ similar to the primary analyses above. Here, however, to determine if GDS was associated with later PACC in the presence of PiB, longitudinal PACC (outcome) was related to longitudinal GDS lagged 1-year backward. For this model, all GDS time points were included, but only PACC values in year 2 onward were included (eFigure 1 in the Supplement). To begin to explore the directionality of the GDS-PACC association, we sought to determine if, conversely, PACC was associated with later GDS in the presence of PiB. We ran the same cross-lagged model as above, reversing the roles of GDS and PACC, such that longitudinal GDS (outcome) was related to longitudinal PACC lagged 1-year backward (eFigure 1 in the Supplement).

#### Sensitivity Analyses

To consider whether GDS items focused on cognitive concerns were driving the association between GDS and PACC, we repeated the primary analyses above with a modified GDS, removing the 4 items related to cognitive concerns from the total score (eAppendix 3 in the Supplement). To examine antidepressant use and depression history as variables associated with GDS, cognition, and amyloid, we introduced antidepressant use (yes or no) or depression history (no history, recent history, or remote history) and their interactions with GDS as covariates in the primary models above.

Finally, to determine if additional AD biomarkers—bilateral hippocampal volume (measured using magnetic resonance imaging, adjusted for intracranial volume) and metabolism in a cortical aggregate of regions associated with MCI and dementia (measured using 18F-fludeoxyglucose PET)^28^ (eAppendix 4 in the Supplement)—moderated the association between depressive symptoms and cognition, the interaction of each of these biomarkers with GDS was separately entered into the primary model above in place of PiB PET. A final model was run that contained all 3 interaction terms (GDS by PiB, GDS by hippocampal volume, and GDS by cortical metabolism).

## Results

The demographics of the study sample at baseline are listed in Table 1. Participants were 164 women and 112 men (mean [SD] age, 73.5 [6.0] years). All participants were cognitively unimpaired, with a global Clinical Dementia Rating of 0 at HABS entry. The mean (SD) GDS at baseline was 3.0 (2.8) (range, 0-12), consistent with HABS entry exclusion criteria. In subsequent years of the study, GDS of the overall sample increased but remained low (eTable 2 and eFigure 2 in the Supplement). Approximately 14.9% (41 of 276) of the sample reported taking antidepressant medication at baseline; 8.0% (22 of 276) reported a depression history with onset in the past 10 years, and 8.0% (22 of 276) reported onset more remotely (Table 1). The mean (SD) baseline PACC was −0.004 (0.67) (range, −2.32 to 1.88), with some participants showing decline on follow-up (eTable 3 and eFigure 3 in the Supplement). The mean (SD) baseline cortical PiB DVR in the sample was 1.16 (0.20) (range, 0.92-1.94). At last follow-up, the mean (SD) GDS was 3.9 (2.9) (range, 0-12), and the mean (SD) PACC was −0.09 (1.27) (range, −5.66 to 1.67).

**Table 1.** Baseline Demographics of the Study Sample Included in the Primary Analyses

Variable	Value
No.	276
Age, mean (SD), y	73.5 (6.0)
Female sex, No. (%)	164 (59.4)
MMSE, mean (SD)	29.0 (1.2)
Education, mean (SD), y	15.8 (3.0)
AMNRT VIQ, mean (SD)	120.8 (9.2)
GDS, mean (SD)	3.0 (2.8)
PiB DVR, mean (SD)	1.16 (0.20)
APOε4 carrier, No. (%)	81 (29.3)
Antidepressant use (yes), No. (%)	41 (14.9)
Depression history, No. (%)	
Yes	44 (15.9)
Recent onset^a^	22 (8.0)
Remote onset^b^	22 (8.0)

Abbreviations: AMNRT VIQ, American National Reading Test Verbal IQ; APOε4, apolipoprotein ε4; GDS, Geriatric Depression Scale; MMSE, Mini-Mental State Examination; PiB DVR, Pittsburgh Compound-B positron emission tomography distribution volume ratio.

^a^ Onset within 10 years of study baseline.

^b^ Onset more than 10 years of study baseline.

### Association Between Longitudinal PACC and Longitudinal GDS

To test our hypothesis of primary interest—that cognition and GDS were inversely associated over the course of the study in the presence of baseline PiB (cortical amyloid)—we used a mixed-effects model with outcome PACC and a longitudinal GDS predictor. We observed a significant (β = −0.19; 95% CI, −0.27 to −0.12; *P* < .001) interaction of baseline PiB retention with longitudinal GDS on PACC decline (Table 2). The association between increasing GDS and PACC decline was only observed at levels of PiB estimated to be equal to 1.06 DVR or greater (Figure). The following were also retained as significant fixed-effects covariates in the final model: baseline age (β = −0.03; 95% CI, −0.04 to −0.02; *P* < .001; older age with lower scores), female sex (β = 0.23; 95% CI, 0.08 to 0.38; *P* < .004; men with lower scores), and education (β = 0.08; 95% CI, 0.05 to 0.10; *P* < .001; less education with lower scores) (Table 2).

**Table 2.** Mixed-Effects Model of PACC Predicted by Longitudinal GDS and Amyloid^a^

Predictor	Partial Unstandardized β (95% CI)^b^	Standardized β	*F* Score	*df*	*P* Value
Longitudinal GDS by PiB interaction	−0.19 (−0.27 to −0.12)	−0.17	26.80	1,907	<.001
Longitudinal GDS	0.20 (0.12 to 0.29)	−0.08	20.80	1,258	<.001
PiB	−0.05 (−0.48 to 0.38)	−0.19	0.05	1,907	.82
Baseline age	−0.03 (−0.04 to −0.02)	−0.24	25.04	1,907	<.001
Female sex	0.23 (0.08 to 0.38)	0.27	8.64	1,271	.004
Education, y	0.08 (0.05 to 0.10)	0.29	36.40	1,907	<.001
Intercept	1.13 (0.01 to 2.25)	−0.19	3.92	1,271	.05

Abbreviations: GDS, Geriatric Depression Scale; PACC, Preclinical Alzheimer Cognitive Composite; PiB, Pittsburgh Compound-B.

^a^ Longitudinal (time-varying) GDS, baseline cortical amyloid (PiB positron emission tomography), age, sex, and education across all time points in the study, listing predictors retained in the final model. *R* = 0.44; *R*^2^ = 0.19; *P* < .001 for actual vs predicted values (fixed). *R* = 0.89; *R*^2^ = 0.79; *P* < .001 for actual vs predicted values (fixed and random). The sample size was 276 participants.

^b^ For binary categorical variables, this is the difference in the means (adjusted for other predictors) relative to the other category.

**Figure.**
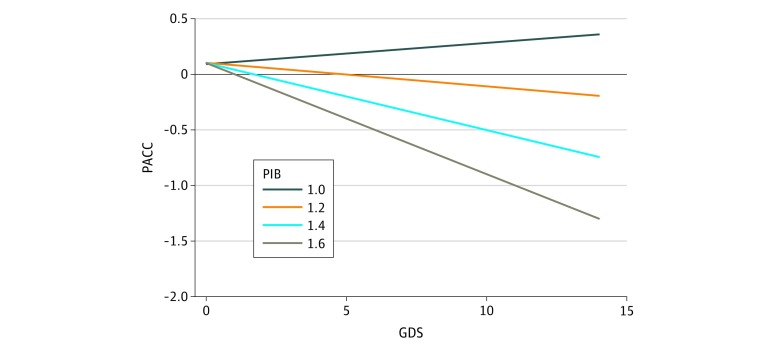
Cortical Amyloid Moderates the Association Between Geriatric Depression Scale (GDS) and Preclinical Alzheimer Cognitive Composite (PACC) Shown is PACC predicted by longitudinal (time-varying) GDS, blind to time. The sample size is 276 participants (1443 observations; 7 maximum observations per participant). Lines are projections used to illustrate associations between GDS and PACC at PiB distribution volume ratio levels 1 SD below the mean (1.0), at the mean (1.2), 1 SD above the mean (1.4), and at the 95th percentile (1.6 because of positive skew). Age and education are set at the means. Sex is female. Only when PiB distribution volume ratio exceeds 1.06 does the association between GDS and PACC become negative. PiB indicates Pittsburgh Compound-B.

To account for the potentially confounding association of time in moderating the association between GDS and PACC, the primary analysis above (a mixed-effects model with outcome PACC and a longitudinal GDS predictor) was repeated adjusting for time, which yielded an essentially equivalent result. These findings are summarized in eTable 4, eAppendix 5, and eFigure 4 in the Supplement.

### Exploratory Analysis of Time Lag Associations

In an exploratory analysis of GDS predicting change in PACC 1 year later, an inverse association moderated by amyloid (PiB retention) was observed, such that greater GDS was associated with lower PACC 1 year later (β = −0.11; 95% CI, −0.18 to −0.05; *P* < .001). The negative association between GDS and PACC occurred at PiB retention levels starting at approximately 1.04 DVR (eTable 5 and eAppendix 6 in the Supplement).

In the reverse cross-lagged analysis, PACC also had a significant inverse association with GDS 1 year later (β = −0.41; 95% CI, −0.71 to −0.10; *P* = .009), but this association was not moderated by PiB retention (eTable 6 in the Supplement). Therefore, in contrast to the association between GDS and 1-year-later PACC, the association of PACC decline with 1-year-later increasing GDS was not dependent on AD pathology.

As in the primary analysis above, exploratory analyses were repeated adjusting for time. This yielded essentially equivalent results.

### Sensitivity Analyses

In separate analyses examining baseline antidepressant use (14.9% [41 of 276] of participants) or depression history (8.0% [22 of 276] with recent onset, 8.0% [22 of 276] with remote onset, and 84.1% [232 of 276] with no history) in the primary models above, in all cases antidepressant use and depression history terms fell out as nonsignificant, leaving results unchanged (eAppendix 3 in the Supplement). This remained the case when these covariates were interacted with time and when a more liberal backward elimination cutoff (*P* = .05) was used.

When investigating associations with additional AD biomarkers (hippocampal volume and cortical metabolism), the interaction between baseline hippocampal volume and longitudinal GDS was marginally associated with PACC decline. When the interaction terms (GDS by PiB, GDS by hippocampal volume, and GDS by cortical metabolism) were entered into the same model, GDS by hippocampal volume was no longer significantly associated with PACC, whereas GDS by PiB remained significantly associated with PACC.

## Discussion

In the present study, we relied on a well-characterized longitudinal cohort of older adults with subthreshold to mild depressive symptoms at study entry. In this cohort, we elucidated the association between changing depressive symptoms and cognition in the presence of baseline cortical amyloid.

We found that increasing depressive symptoms were associated with worsening cognition, an association that occurred at amyloid (PiB retention) above 1.06 DVR. This supports a model in which shared pathological processes, particularly cortical amyloid, might underlie both depression and cognitive decline in preclinical AD, thus explaining their association through the course of the study. We observed this association at PiB levels in a range below the published threshold of 1.20 DVR that marks amyloid positivity in the HABS cohort,^25^ suggesting that the association between depressive symptoms and cognition is one that occurs at early stages of the AD pathological spectrum. This also supports the potential utility of rising depressive symptoms as a marker of changing cognition that could potentially facilitate early detection of at-risk individuals. In addition, our results suggest that depressive symptoms in combination with cognitive measures could be viable outcomes in trials aimed at preventing the clinical symptoms of AD.

Our analyses examined associations among depressive symptoms and cognition as moderated by amyloid, rather than casual associations. Therefore, while results suggest the viability of depressive symptoms as outcomes in AD clinical trials, it remains unclear whether intervention on depressive symptoms would improve cognitive function (and vice versa). Future prospective interventional studies targeting depressive symptoms in cognitively normal older adults are needed to provide more definitive support for this strategy. In addition, while elevated amyloid may increase the risk for cognitive decline, not all individuals with depressive symptoms and cortical amyloid will experience progressive cognitive decline. Rather, this association may be influenced by additional risk and protective factors beyond the scope of our analyses, which is an important area of future investigation. Indeed, our finding that baseline hippocampal volume moderates the longitudinal association between depressive symptoms and cognition supports a role for related pathways, which could include tau-mediated neurodegeneration, hypercortisolemia, or inflammation as potential intervention targets.

Our findings extend previous work studying the association among depressive symptoms, cognition, and risk for AD clinical progression.^11,29,30,31^ Across separate cohorts of cognitively normal older individuals (n = 1764) from Rush University, depressive symptoms averaged across time were associated with more rapid cognitive decline and progression from cognitively normal status to MCI.^29^ In the 582 individuals for whom postmortem data were available, the association between depressive symptoms and cognitive decline was not moderated by neuropathology, including β-amyloid plaques.^29^ Depressive symptoms in this sample from Rush were in the low range at study entry, and overall follow-up time was longer and included individuals who progressed to MCI and dementia. This may in part account for the differences in our findings in that we focused on cognitively unimpaired older adults, a small fraction of whom (6.5% [18 of 276]) progressed to MCI over follow-up. In addition, our in vivo continuous measure of cortical amyloid at baseline, rather than β-amyloid plaque count at the final time point, as was the case in the postmortem data,^29^ may have allowed for a more sensitive temporal assessment of time point to time point change in clinical symptoms in relation to AD pathology.

Findings from exploratory cross-lagged models showed that the interaction between cortical amyloid and greater depressive symptoms was associated with lower cognitive performance 1 year later. Conversely, lower cognitive performance was associated with higher GDS 1 year later, but this latter association was not moderated by amyloid. Together, these findings are consistent with the hypothesis that depressive symptoms may be an early behavioral manifestation of AD and may mark (or even hasten) subsequent cognitive decline. While these exploratory results must be interpreted cautiously, they suggest that different processes, biological vs psychological, could moderate the association between early depression and later cognitive performance compared with early cognitive decline and later depression. Further work in larger prospective studies is needed to deconstruct the temporal ordering of these associations and to inform early detection and intervention in individuals at the highest level of risk.

The associations among depression, cognition, and amyloid persisted even when examining a GDS without the 4 cognitive items and when adjusting for baseline antidepressant use and depression history. Although we cannot rule out that individuals reporting depressive symptoms also had cognitive concerns, sensitivity analyses suggested that our GDS findings were not driven by items that probe cognitive concerns.

### Strengths and Limitations

Our study has a number of strengths and limitations. Strengths included analyzing data from a well-characterized cohort with detailed longitudinal measures of depression, cognition, and baseline amyloid PET imaging. Although the limited range of depressive symptoms may have influenced the magnitude of our findings, it also allowed for a focused study of subthreshold to mild depressive symptoms, which are prevalent among older individuals and may more closely relate to cognitive trajectory than more severe depressive symptoms.^30^ While depression was captured by the GDS self-report, rather than a structured psychiatric interview, the GDS has clinical relevance because it is similar to depression assessments in routine clinical encounters and in clinical trials. We focused on cortical amyloid, although a number of other biological processes have been associated with depression and cognition in late life, including vascular disease, inflammation, neuronal network disruption, and tau-mediated neurodegeneration,^6,32,33,34,35^ all of which will be important to examine in future work. In addition, other time-varying factors, such as antidepressant treatment adherence, cognitive and social activities, and medical comorbidity, although beyond the scope of the present analyses, may have influenced depressive symptoms and cognitive function. Our convenience sample was educated, predominantly of white race/ethnicity, and in overall good physical health with low vascular disease burden,^36^ underscoring the importance of replicating our findings in population-based samples.

## Conclusions

Our findings add to a growing body of literature supporting an association between concurrent changes in depression and cognition, providing some of the first focused evidence of this association in preclinical AD defined based on amyloid PET neuroimaging. Results support depressive symptoms as among the earliest changes related to AD pathological burden and cognitive decline. They underscore the importance of close monitoring of older adults with depressive symptoms in clinical settings and the need for further study of the potential utility of targeting these symptoms in preclinical AD intervention studies aimed at mitigating cognitive decline.

## Notes

Supplement. **eTable 1.** Number of Participants (Frequency, Percent, Cumulative Frequency and Cumulative Percent) With a Given Number of Follow-up Visits, Ranging From 2 to 7 Years **eTable 2.** Average Geriatric Depression Scale (GDS) Score by Year in the Study **eTable 3.** Average PACC Score by Year in the Study **eTable 4.** Mixed-Effects Model of PACC (Preclinical Alzheimer Cognitive Composite) Predicted by Cortical Amyloid (PiB PET) and Longitudinal GDS (Geriatric Depression Scale) Displaying Predictors Retained in the Final Model, Including Adjustment for Time **eTable 5.** Mixed-Effects Model of PACC (Preclinical Alzheimer Cognitive Composite) Predicted by 1-Year Lagged Longitudinal GDS (Geriatric Depression Scale), Baseline Cortical Amyloid (PiB PET), Age, Sex, Education, Across All Time Points in the Study Displaying Predictors Retained in the Final Model **eTable 6.** Mixed-Effects Model of GDS (Geriatric Depression Scale) Predicted by 1-Year Lagged Longitudinal PACC (Preclinical Alzheimer Cognitive Composite), Baseline Cortical Amyloid (PiB PET), Age, Sex, Education, Across All Time Points in the Study, Displaying Predictors Retained in the Final Model **eAppendix 1.** Depression Assessment **eAppendix 2.** Statistical Methods **eAppendix 3.** Sensitivity Analyses **eAppendix 4.** Evaluation of Additional Biomarkers **eAppendix 5.** Analyses Adjusting for Time **eAppendix 6.** Exploratory Analysis of Time Lag Effects **eFigure 1.** Path Diagram Representation of Cross-Lagged Effects **eFigure 2.** Spaghetti Plot of GDS vs Time **eFigure 3.** Spaghetti Plot of PACC vs Time **eFigure 4.** Cortical Amyloid Moderates the Association Between GDS and PACC Including Adjustment for Time, Holding Time and All Predictors Constant **eReferences.**
