# Supplementary material for: Longitudinal Association of Depression Symptoms With Cognition and Cortical Amyloid Among Community-Dwelling Older Adults
Source: JAMA Netw Open. 2019 Aug 9;2(8):e198964. doi: 10.1001/jamanetworkopen.2019.8964 (PMC6692684; doi:10.1001/jamanetworkopen.2019.8964)
Supplement: Supplement. — eTable 1. Number of Participants (Frequency, Percent, Cumulative Frequency and Cumulative Percent) With a Given Number of Follow-up Visits, Ranging From 2 to 7 Years eTable 2. Average Geriatric Depression Scale (GDS) Score by Year in the Study eTable 3. Average PACC Score by Year in the Study eTable 4. Mixed-Effects Model of PACC (Preclinical Alzheimer Cognitive Composite) Predicted by Cortical Amyloid (PiB PET) and Longitudinal GDS (Geriatric Depression Scale) Displaying Predictors Retained in the Final Model, Including Adjustment for Time eTable 5. Mixed-Effects Model of PACC (Preclinical Alzheimer Cognitive Composite) Predicted by 1-Year Lagged Longitudinal GDS (Geriatric Depression Scale), Baseline Cortical Amyloid (PiB PET), Age, Sex, Education, Across All Time Points in the Study Displaying Predictors Retained in the Final Model eTable 6. Mixed-Effects Model of GDS (Geriatric Depression Scale) Predicted by 1-Year Lagged Longitudinal PACC (Preclinical Alzheimer Cognitive Composite), Baseline Cortical Amyloid (PiB PET), Age, Sex, Education, Across All Time Points in the Study, Displaying Predictors Retained in the Final Model eAppendix 1. Depression Assessment eAppendix 2. Statistical Methods eAppendix 3. Sensitivity Analyses eAppendix 4. Evaluation of Additional Biomarkers eAppendix 5. Analyses Adjusting for Time eAppendix 6. Exploratory Analysis of Time Lag Effects eFigure 1. Path Diagram Representation of Cross-Lagged Effects eFigure 2. Spaghetti Plot of GDS vs Time eFigure 3. Spaghetti Plot of PACC vs Time eFigure 4. Cortical Amyloid Moderates the Association Between GDS and PACC Including Adjustment for Time, Holding Time and All Predictors Constant eReferences. [file jamanetwopen-2-e198964-s001.pdf]

Supplementary Online Content

Gatchel JR, Rabin JS, Buckley RF, et al; Harvard Aging Brain Study. Longitudinal association of depression symptoms with cognition and cortical amyloid among community-dwelling older adults. *JAMA Netw Open*. 2019;2(8):e198964. doi:10.1001/jamanetworkopen.2019.8964

eTable 1. Number of Participants (Frequency, Percent, Cumulative Frequency and Cumulative Percent) With a Given Number of Follow-up Visits, Ranging From 2 to 7 Years
eTable 2. Average Geriatric Depression Scale (GDS) Score by Year in the Study
eTable 3. Average PACC Score by Year in the Study
eTable 4. Mixed-Effects Model of PACC (Preclinical Alzheimer Cognitive Composite) Predicted by Cortical Amyloid (PiB PET) and Longitudinal GDS (Geriatric Depression Scale) Displaying Predictors Retained in the Final Model, Including Adjustment for Time
eTable 5. Mixed-Effects Model of PACC (Preclinical Alzheimer Cognitive Composite) Predicted by 1-Year Lagged Longitudinal GDS (Geriatric Depression Scale), Baseline Cortical Amyloid (PiB PET), Age, Sex, Education, Across All Time Points in the Study Displaying Predictors Retained in the Final Model
eTable 6. Mixed-Effects Model of GDS (Geriatric Depression Scale) Predicted by 1-Year Lagged Longitudinal PACC (Preclinical Alzheimer Cognitive Composite), Baseline Cortical Amyloid (PiB PET), Age, Sex, Education, Across All Time Points in the Study, Displaying Predictors Retained in the Final Model
eAppendix 1. Depression Assessment
eAppendix 2. Statistical Methods
eAppendix 3. Sensitivity Analyses
eAppendix 4. Evaluation of Additional Biomarkers
eAppendix 5. Analyses Adjusting for Time
eAppendix 6. Exploratory Analysis of Time Lag Effects
eFigure 1. Path Diagram Representation of Cross-Lagged Effects
eFigure 2. Spaghetti Plot of GDS vs Time
eFigure 3. Spaghetti Plot of PACC vs Time
eFigure 4. Cortical Amyloid Moderates the Association Between GDS and PACC Including Adjustment for Time, Holding Time and All Predictors Constant
eReferences.

This supplementary material has been provided by the authors to give readers additional information about their work.

© 2019 Gatchel JR et al. *JAMA Network Open*.

eTable 1. Number of Participants (Frequency, Percent, Cumulative Frequency and Cumulative Percent) With a Given Number of Follow-up Visits, Ranging From 2 to 7 Years

Number of visits	Frequency	Percent	Cumulative Frequency	Cumulative Percent
2	11	3.99	11	3.99
3	11	3.99	22	7.97
4	61	22.10	83	30.07
5	73	26.45	156	56.52
6	53	19.20	209	75.72
7	67	24.28	276	100.00

© 2019 Gatchel JR et al. *JAMA Network Open*.

eTable 2. Average Geriatric Depression Scale (GDS) Score by Year in the Study

Year in study	N	Mean +/- Standard deviation	Range
1	276	3.0 +/- 2.8	(0-12)
2	275	3.9 +/- 3.9	(0-21)
3	264	3.9 +/- 3.9	(0-21)
4	251	4.0 +/- 4.1	(0-21)
5	192	4.1 +/- 4.2	(0-24)
6	119	3.8 +/- 3.0	(0-15)
7	66	3.9 +/- 2.9	(0-12)

© 2019 Gatchel JR et al. *JAMA Network Open*.

eTable 3. Average PACC Score by Year in the Study

Year in study	N	Mean +/- Standard deviation	Range
1	276	-0.004 +/- 0.67	(-2.32 ,1.88)
2	276	-0.04 +/- 0.74	(-3.17, 2.04)
3	264	-0.05 +- 0.78	(-5.34, 2.19)
4	254	0.11 +/- 0.81	(-3.11, 2.19)
5	194	0.048 +/- 0.87	(-3.59, 2.02)
6	120	0.04 +/- 0.91	(-3.36, 1.90)
7	67	-0.09 +/- 1.27	(-5.66, 1.67)

© 2019 Gatchel JR et al. *JAMA Network Open*.

eTable 4. Mixed-Effects Model of PACC (Preclinical Alzheimer Cognitive Composite) Predicted by Cortical Amyloid (PiB PET) and Longitudinal GDS (Geriatric Depression Scale) Displaying Predictors Retained in the Final Model, Including Adjustment for Time[a]. Model: R =0.46, R^2 =0.21, p<0.001 for actual vs. predicted values (fixed); R=0.93, R^2= 0.86, p<.001 for actual vs. predicted values (fixed and random). PiB= Pittsburgh Compound B; CI= Confidence Interval; Df= degrees of freedom.

Predictor	Partial Unstandardized B[b]	95% CI for B	Standardized B	F	Df	P
Longitudinal GDS X PiB	-0.14	(-0.18, -0.089)	-0.12	32.65	(1, 887)	<0.001
PiB X $Time^2$ (quadratic)	-0.049	(-0.079, -0.018)	-0.012	9.68	(1, 887)	0.002
Longitudinal GDS	0.15	(0.093, 0.21)	-0.037	26.77	(1, 887)	<0.001
PiB	0.23	(-0.20, 0.65)	-0.071	1.11	(1, 887)	0.29
Sex (Female)	0.26	(0.11, 0.40)	0.32	12.37	(1, 271)	p<0.001
Baseline Age	-0.024	(-0.036, -0.013)	-0.18	16.56	(1, 887)	<0.001
Education (years)	0.077	(0.054, 0.10)	0.28	42.05	(1, 887)	<0.001
$Time^2$ (quadratic)	0.045	(0.009, 0.081)	-0.014	6.01	(1, 887)	0.014

[a]Analytic Sample Size=276
[b]For binary categorical variables, this is the difference in means (adjusted for other predictors) of the category in parentheses relative to the other category.

© 2019 Gatchel JR et al. *JAMA Network Open*.

eTable 5. Mixed-Effects Model of PACC (Preclinical Alzheimer Cognitive Composite) Predicted by 1-Year Lagged Longitudinal GDS (Geriatric Depression Scale), Baseline Cortical Amyloid (PiB PET), Age, Sex, Education, Across All Time Points in the Study Displaying Predictors Retained in the Final Model[a]. Model: R =0.45, R^2 =0.20, p<0.001 for actual vs. predicted values (fixed); R=0.88, R^2= 0.77, p<0.001 for actual vs. predicted values (fixed and random). PiB= Pittsburgh Compound B; CI= Confidence Interval; Df= degrees of freedom.

**Predictor**	**Partial Unstandardized B[b]**	**95% CI for B**	**Standardized B**	**F**	**Df**	**P**
Longitudinal GDS-1 year lagged X PIB	-0.11	(-0.18, -0.045)	-0.084	10.74	(1, 650)	0.001
Baseline age	-0.034	(-0.047, -0.02)	-0.20	24.45	(1, 650)	<0.001
Sex (female)	0.22	(0.059, 0.39)	0.22	7.16	(1, 258)	0.008
Education (years)	0.07	(0.04, 0.09)	0.20	25.02	(1, 650)	<0.001
Longitudinal GDS-1 year lagged	0.12	(0.036, 0.20)	-0.048	7.98	(1, 240)	0.005
PiB	-0.61	(-1.09, -0.13)	-0.21	6.13	(1, 650)	0.014
Intercept	2.02	(0.82, 3.22)	-0.14	10.96	(1, 258)	0.001

[a]Analytic sample size=263
[b]For binary categorical variables, this is the difference in means (adjusted for other predictors) of the category in parentheses relative to the other category.

© 2019 Gatchel JR et al. *JAMA Network Open*.

eTable 6. Mixed-Effects Model of GDS (Geriatric Depression Scale) Predicted by 1-Year Lagged Longitudinal PACC (Preclinical Alzheimer Cognitive Composite), Baseline Cortical Amyloid (PiB PET), Age, Sex, Education, Across All Time Points in the Study, Displaying Predictors Retained in the Final Model. Model: R =0.13, R^2 =0.02, p<0.001 for actual vs. predicted values (fixed); R=0.89, R^2= 0.78, p<0.001 for actual vs. predicted values (fixed and random). PiB= Pittsburgh Compound B; CI= Confidence Interval; Df= degrees of freedom.

**Predictor**	**Partial Unstandardized B**	**95% CI for B**	**Standardized B**	**F**	**Df**	**P**
Longitudinal PACC-1 year lagged	-0.41	(-0.71, -0.10)	-0.30	6.82	(1, 898)	0.009
Intercept	3.98	(3.57, 4.40)	3.97	356.08	(1, 265)	<0.001

[a]Analytic sample size=266.

© 2019 Gatchel JR et al. *JAMA Network Open*.

eAppendix 1. Depression Assessment
After performing correction for missing data, only 0.6 percent of GDS total scores (8 out of 1451 entries) were missing across all longitudinal visits). For all primary analyses, the GDS total score was used.

eAppendix 2. Statistical Methods
For all models, residuals from the final selected fixed effects and mixed effects models were examined for conformance to significance test assumptions of normality and homoscedasticity.

eAppendix 3. Sensitivity Analyses
To examine the potential confound of four GDS items that probe cognitive concerns, we calculated an adjusted GDS score excluding four items :"Is your mind as clear as it used to be;" "Do you have trouble concentrating;" "Is it easy for you to make decisions;" and "Do you feel you have more problems with memory than most?" We repeated analyses with GDS scores computed to remove the contribution of 4 items that assess cognitive-related symptoms (calculated as described above). There was no appreciable change in results using this adjusted GDS measure (data not shown), not surprising given that the adjusted GDS was highly correlated with the total GDS score (r=0.97 across all visit records).

eAppendix 4. Evaluation of Additional Biomarkers
Hippocampal volume measurement was derived from structural magnetic resonance imaging (MRI) data as described previously[1]. MRI data data were collected on a Siemens TrioTim 3-T scanner; an MPRAGE sequence was used to acquire high-resolution T1-weighted anatomical images[1]. Estimation of hippocampal volume was derived using FreeSurfer 6 (http://surfer.nmr.mgh.harvard.edu), as described previously[1]. Briefly, measures of bilateral hippocampal volume, collapsed across right and left hemispheres, were regressed across intracranial volume[1].

18-F-Fluorodeoxygluocse (FDG) positron emission tomography (PET) data were acquired as described previously[1]. PET data sets were co-registered to a participant's MRI data using SPM12. FDG PET values were expressed as standardized uptake volume ratios, with normalization to the cerebellar gray for each participant. An association cortex region of interest was derived from an aggregate of regions—bilateral precuneus, inferior parietal and inferior temporal cortices—previously found to be altered in mild cognitive impairment (MCI) and in cognitively normal individuals with high cortical amyloid[1,2]. Adjustments were made, as described previously, to take into account the impact of underlying anatomical variations on FDG PET values[1].

eAppendix 5. Analyses Adjusting for Time
We repeated primary analyses holding time constant (introducing fixed terms for linear and quadratic years in the study). The results of our primary analyses were largely unchanged (eTable 4, and eFigure 4).

eAppendix 6. Exploratory Analysis of Time-Lagged Associations
Although the cross-lag approach we used in exploratory analyses may be confounded by different test/retest reliabilities for the two variables being compared, for our two variables of primary interest, PACC and GDS, test/retest reliabilities were comparable (data not shown).

© 2019 Gatchel JR et al. *JAMA Network Open*.

eFigure 1. Path Diagram Representation of Cross-Lagged Effects

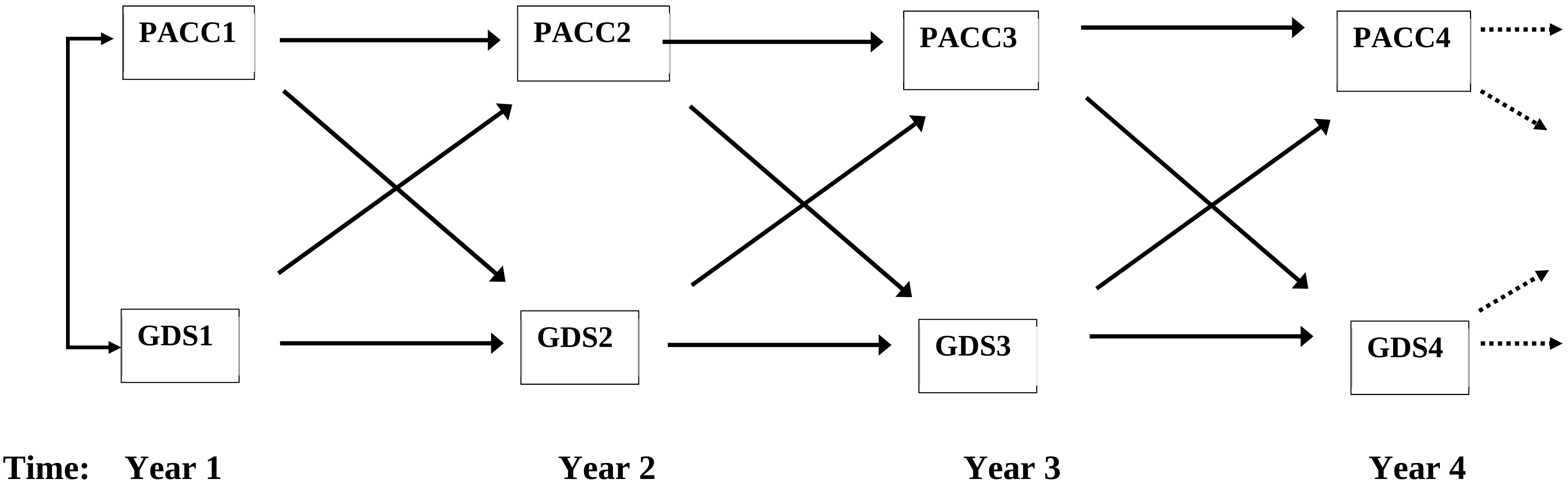

eFigure 1. Path Diagram Representation of Cross Lagged Effects. Solid black arrows indicate one-year lagged effects, dotted lines indicate further extensions into the future. The double-headed arrow indicates an initial correlation. “PACC(n)” = Preclinical AD Cognitive Composite assessed at year “n”; “GDS(n)” = Geriatric Depression Scale assessed at year “n”. For simplicity, possible effects across more than 1 year are not indicated).

© 2019 Gatchel JR et al. *JAMA Network Open*.

eFigure 2. Spaghetti Plot of GDS vs Time

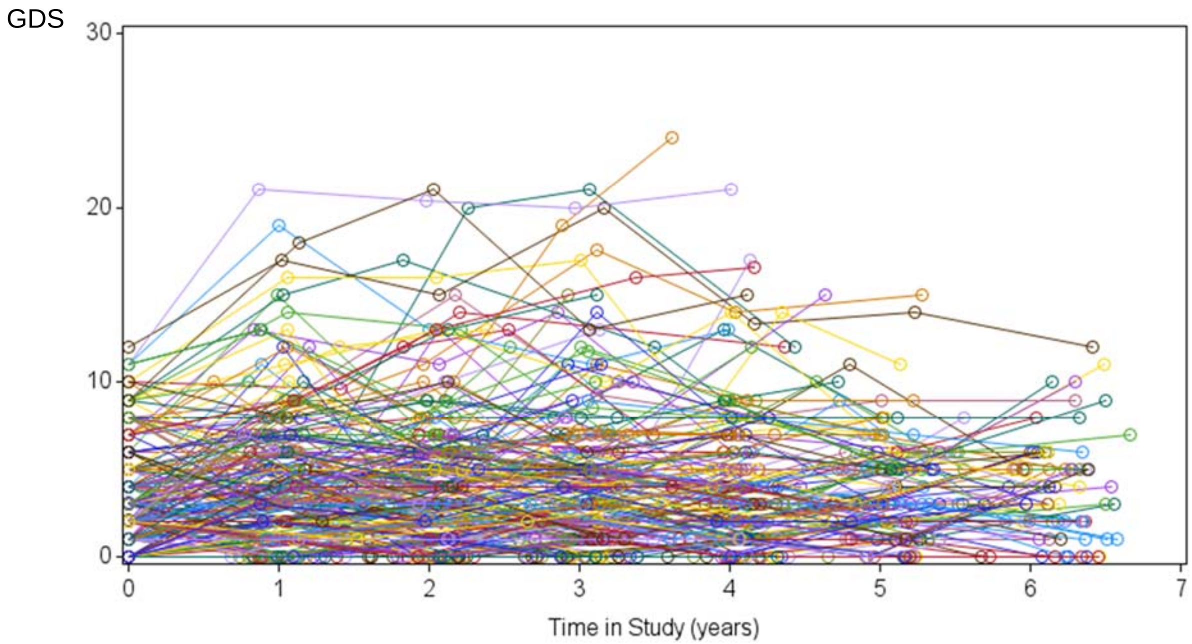

eFigure 2. “Spaghetti” longitudinal plot of GDS (Geriatric Depression Scale) versus time in the study. (Lines connect scores for the same participant).

© 2019 Gatchel JR et al. *JAMA Network Open*.

eFigure 3. Spaghetti Plot of PACC vs Time

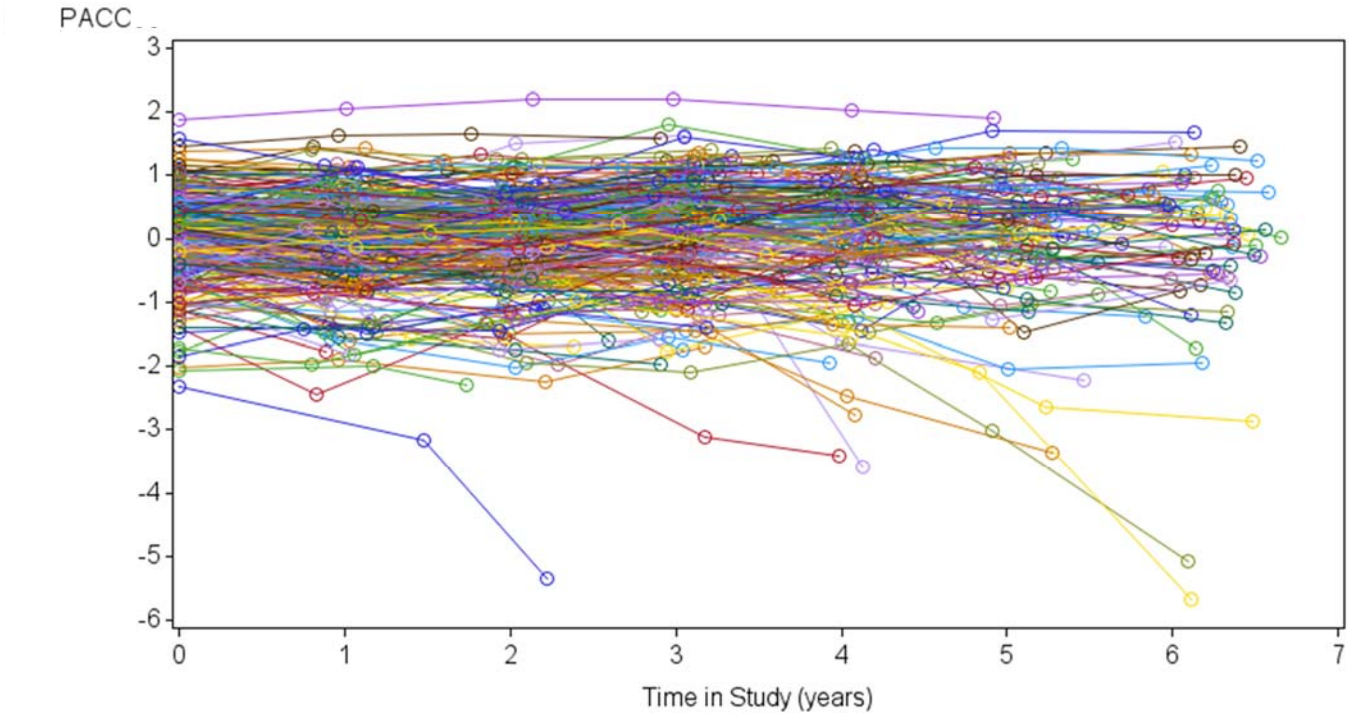

eFigure 3: “Spaghetti” longitudinal plot of PACC (Preclinical Alzheimer Cognitive Composite) versus time in the study. (Lines connect scores for the same participant).

© 2019 Gatchel JR et al. *JAMA Network Open*.

eFigure 4. Cortical Amyloid Moderates the Association Between GDS and PACC Including Adjustment for Time, Holding Time and All Predictors Constant

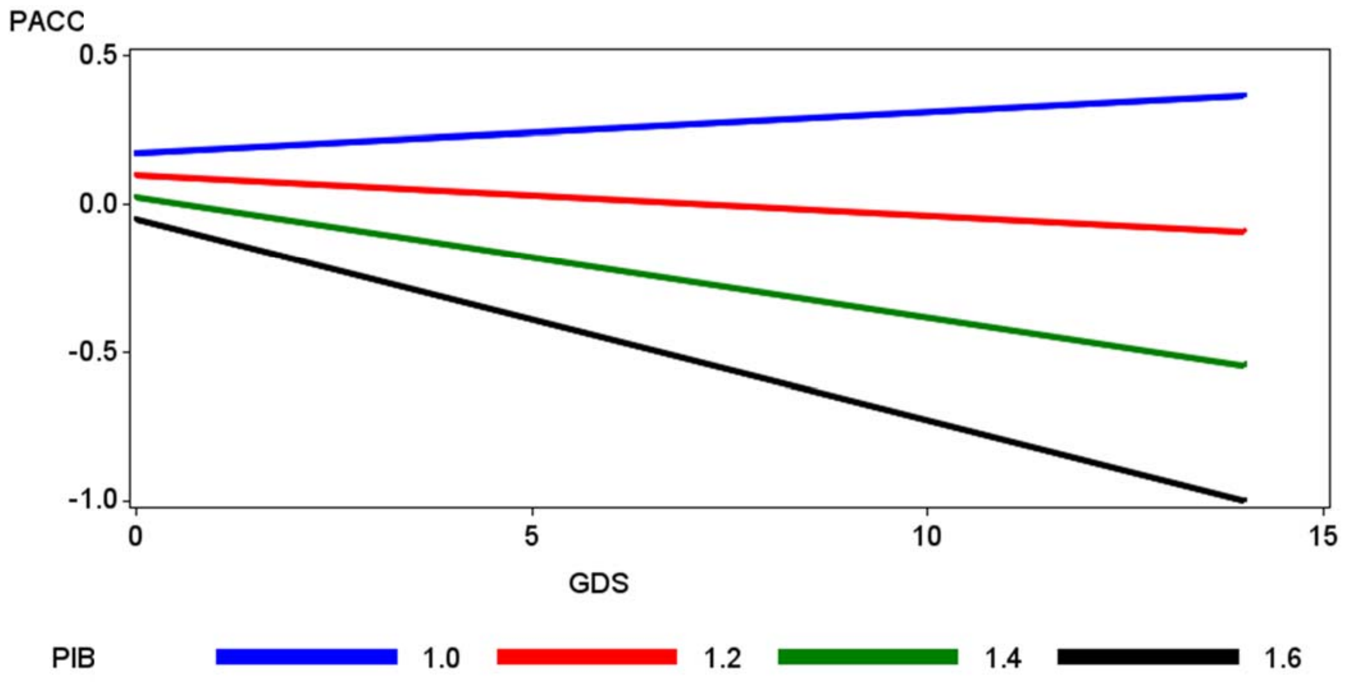

eFigure 4. PACC predicted by longitudinal GDS, including adjustment for time, holding time and all predictors constant. Sample size: n=276 participants. Lines are projections used to illustrate associations between GDS and PACC at PiB DVR levels 1 SD below the mean (1), mean (1.2), 1 SD above mean (1.4), and 95th %tile (1.6 because of positive skew). Time, age, and education are set at means. Sex = female. Only when PiB is > 1.1 does the association between GDS and PACC become negative. PACC= Preclinical Alzheimer Cognitive Composite; GDS= Geriatric Depression Scale; PIB: Pittsburgh Compound B; DVR: Distribution Volume Ratio; SD: standard deviation.

© 2019 Gatchel JR et al. *JAMA Network Open*.

eReferences.

1. Hanseeuw BJ, Schultz AP, Betensky RA, Sperling RA, Johnson KA. Decreased hippocampal metabolism in high-amyloid mild cognitive impairment. *Alzheimers Dement.* 2016;12(12):1288-1296.

2. Herholz K. Cerebral glucose metabolism in preclinical and prodromal Alzheimer's disease. *Expert Rev Neurother.* 2010;10(11):1667-1673.

© 2019 Gatchel JR et al. *JAMA Network Open*.
